# Immune system changes during simulated planetary exploration on Devon Island, high arctic

**DOI:** 10.1186/1471-2172-8-7

**Published:** 2007-05-23

**Authors:** Brian Crucian, Pascal Lee, Raymond Stowe, Jeff Jones, Rainer Effenhauser, Raymond Widen, Clarence Sams

**Affiliations:** 1 Wyle Laboratories/NASA-JSC, 1290 Hercules Drive, Houston, Texas, 77058, USA; 2 Mars Institute, SETI Institute & NASA Ames Research Center, 515 N. Whisman Road, Moffett Field, CA, 94043, USA; 3 Microgen Laboratories, 903 Texas Avenue, La Marque, Texas, 77568, USA; 4 NASA-JSC, NASA Parkway, Houston, Texas, 77058, USA; 5 Tampa General Hospital, PO Box 1289, Tampa, Florida, 33601, USA

## Abstract

**Background:**

Dysregulation of the immune system has been shown to occur during spaceflight, although the detailed nature of the phenomenon and the clinical risks for exploration class missions have yet to be established. Also, the growing clinical significance of immune system evaluation combined with epidemic infectious disease rates in third world countries provides a strong rationale for the development of field-compatible clinical immunology techniques and equipment. In July 2002 NASA performed a comprehensive immune assessment on field team members participating in the Haughton-Mars Project (HMP) on Devon Island in the high Canadian Arctic. The purpose of the study was to evaluate the effect of mission-associated stressors on the human immune system. To perform the study, the development of techniques for processing immune samples in remote field locations was required. Ten HMP-2002 participants volunteered for the study. A field protocol was developed at NASA-JSC for performing sample collection, blood staining/processing for immunophenotype analysis, whole-blood mitogenic culture for functional assessments and cell-sample preservation on-location at Devon Island. Specific assays included peripheral leukocyte distribution; constitutively activated T cells, intracellular cytokine profiles, plasma cortisol and EBV viral antibody levels. Study timepoints were 30 days prior to mission start, mid-mission and 60 days after mission completion.

**Results:**

The protocol developed for immune sample processing in remote field locations functioned properly. Samples were processed on Devon Island, and stabilized for subsequent analysis at the Johnson Space Center in Houston. The data indicated that some phenotype, immune function and stress hormone changes occurred in the HMP field participants that were largely distinct from pre-mission baseline and post-mission recovery data. These immune changes appear similar to those observed in astronauts following spaceflight.

**Conclusion:**

The immune system changes described during the HMP field deployment validate the use of the HMP as a ground-based spaceflight/planetary exploration analog for some aspects of human physiology. The sample processing protocol developed for this study may have applications for immune studies in remote terrestrial field locations. Elements of this protocol could possibly be adapted for future in-flight immunology studies conducted during space missions.

## Background

The growing diagnostic significance of clinical immunology combined with epidemic microbial disease rates in third-world countries make it clear that improved field-laboratory capabilities may be needed in the future to supportimmune testing or research. Whereas technological advances have already made it possible to provide field-testing for other laboratory disciplines (chemistry, hematology, etc.), field immunology research has remained a challenge. This is due in part to the labile nature of cytokines and chemokines, the requirement for high-quality cellular samples with surface antigen integrity intact, and in some cases the need for live cell cultures. The size, weight and power requirements of most of the required instrumentation (incubators, flow cytometers, etc.) also largely preclude their routine use in the field.

Dysregulation of the immune system has been documented to occur during spaceflight. There have been several excellent reviews published regarding this phenomenon [1-4]. Specific post-flight or in-flight changes observed include alterations in cytokine production patterns [5-14], NK cell function [15-17], leukocyte distribution [6,18], reactivation of latent herpes viruses [19-22], monocyte function [23,24], neutrophil function [25,18], T cell intracellular signaling [26-30], neuorendocrine responses [31] and leukocyte proliferation following activation [32,33]. Spaceflight-associated immune dysfunction may be due to flight-related factors (microgravity, fluid shifts, radiation) or mission-associated factors (confinement, isolation, physiologic stress, nutrition, altered circadian rhythms, altered microbial environment, etc.) not uniquely associated with spaceflight. NASA is currently performing studies to investigate the causes and clinical risk associated with prolonged spaceflight-associated immune dysregulation in astronauts, prior to the initiation of exploration class missions. Aside from microgravity, many of the challenges in performing in-flight immune studies are similar to those faced by personnel performing clinical medicine in remote field locations or third-world countries. These challenges include isolation, difficulty in transporting laboratory equipment, power requirements, reagent stability, as well as the integrity of processed and stored biological samples.

To evaluate the effects of mission-associated factors on human physiology, ground-based 'spaceflight analogs' may be used [34]. A variety of analogs are available, each unique and exerting some influence on human physiology that is similar to one (or more) aspects of space flight. For ground-based studies, it is very important to choose the analog that is most appropriate for the physiological system of interest. Examples of such analogs are extended bed rest (for fluid shifts, bone and muscle loss), closed chamber confinement (for psychological and isolation issues) and Antarctic winter-over (for isolation, confinement and stress). An excellent ground based flight analog for immune studies would simulate mission-associated stress, isolation and disrupted circadian rhythms. During Antarctic winter-over, subjects experience prolonged isolation in a harsh extreme environment, and several comprehensive immune studies have been conducted during these expeditions. Antarctic immune studies have shown decreased cell mediated immune responses [35-37], reduced T cell function [38], altered cytokine production profiles [38-40] and latent herpesvirus reactivation [37,38]. A study of antibody production following immunization during winter over found no alterations mid mission [41], potentially indicating humoral immunity is unchanged in the presence of altered cellular immunity. Thus, the Antarctic analog has been the most successful to date in simulating spaceflight-associated immune dysregulation.

**Figure 1:**
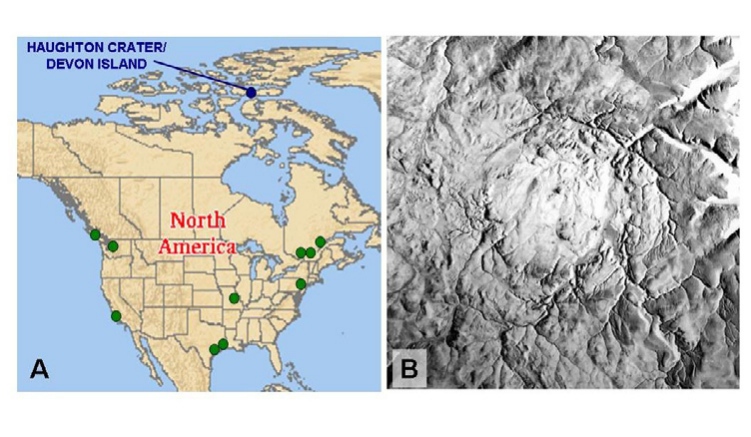
(A) Map indicating the location of the HMP research Station on Devon Island, Canadian High Arctic. The home locations of the 10 HMP study participants are also indicated (green circles). (B) Airborne synthetic aperture radar image of Haughton Crater acquired in 1998 by the Intera STAR X-band radar system. The width of the scene is 36 km. The crater is approximately 20 km wide. North is up. Image courtesy Geological Survey of Canada and HMP.

**Figure 2:**
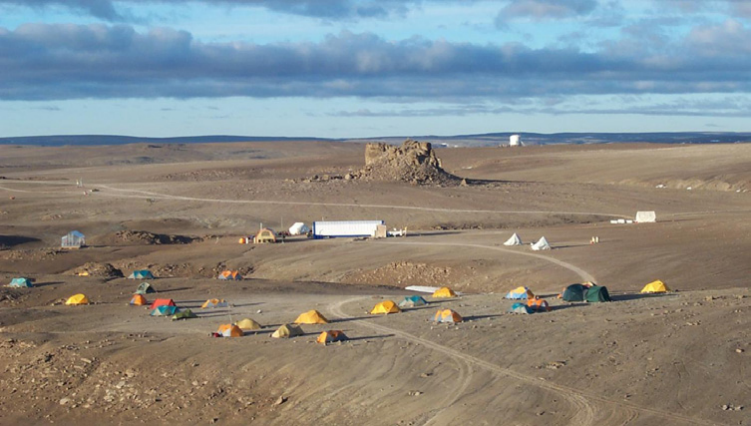
Overview photo showing the HMP Research Station and surrounding area at the time the study was conducted (Summer, 2002). The tent area in the foreground served as personal living quarters for HMP field participants. Immediately behind is the central base camp, consisting of core facilities, laboratory space, communications and mess tent, etc. The prototype Mars greenhouse is located to the left of the core facility. The rim of the impact crater is distant-right. The FMARS habitat, located on the crater rim, was not used in this study.

The NASA Haughton-Mars Project (HMP) is an international field research project centered on the scientific study of the Haughton meteorite impact structure and surrounding terrain on Devon Island, Nunavut Territory, Canadian High Arctic. The HMP is viewed as an analog for planetary exploration, in particular for exploration of the Moon and Mars (figure 1) [42]. Haughton crater is the only impact crater on Earth set in a true *polar desert *(figure 2): an environment that is cold (annual average T is -16°C), dry (annual precipitation is < 13 mm), windy, rocky, sparsely vegetated, and receiving a high solar UV flux in the summer. The HMP supports an exploration program focused on defining the requirements, and on developing the technologies and strategies, needed for planning future robotic and human planetary missions. The HMP is particularly well suited for exploration-related human physiology studies because field personnel are subject to actual *and *relevant environmental stressors, although clearly not as extreme as those encountered in space. In addition, they are engaged in field exploration tasks that in many cases are direct analogs to those anticipated for the Moon and Mars. Activities at HMP are driven by actual field exploration and research agendas, not simulations thereof, which makes the surface operations, lessons learned and the human experience acquired particularly valuable. The following factors encountered by HMP field participants are particularly relative to spaceflight/planetary exploration:

• Long travel to and from Devon Island (several days of travel followed by weeks of stay)

• Relatively harsh polar desert environment

• Disrupted circadian rhythms (24 hours of daylight during the Summer field season)

• Relative isolation from the outside world (with limited exception)

• Limited local infrastructure (HMP Research Station is analogous to early lunar or Martian outpost)

• Moon and Mars-relevant (analog) exploration/field/EVA activities

• Reliance on remote telemedicine and communications equipment.

These factors make the HMP potentially a good analog for spaceflight-associated immune dysfunction studies. The objectives of the present study were to (1) develop and field-evaluate methods for processing biological samples to support immune function testing in remote locations; and (2) utilize the protocol to assess mission-associated immune changes during a HMP mission. These methods could be adapted for monitoring the immune system of astronauts during exploration-class spaceflight or for immunology research studies in third-world/field locations.

**Figure 3:**
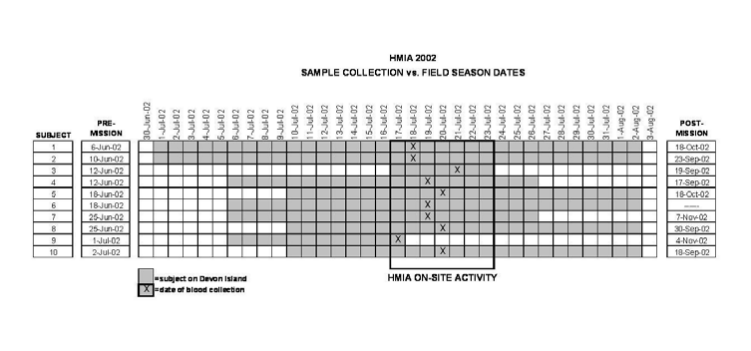
Sample collection dates vs. field season activity for all ten study subjects. Participants are on-location at Devon Island for varying lengths of time, depending on the individual subject's mission objectives. In general, the field season lasts for approximately one month each summer. Sample consisted of 20 ml whole blood. Pre- and post-mission samples were collected at local clinics and overnight shipped to JSC for analysis. Field samples were processed on-location (including cell culture) and stabilized for transport to JSC.

For this study, a deployment to the HMP Research Station was carried out in July 2002, during which a mobile biological sample processing facility was established in the station's laboratory tent facility. A panel of assays was developed that would comprehensively assess for each participant: (1) the presence or absence of specific immune responses; (2) the functional responsiveness immune system; (3) the presence or absence of latent EBV reactivation; (4) physiological stress. Ten subjects deployed to Devon Island during the 2002 field season participated in the study (figure 3). The specific assays were as follows:

1. Peripheral blood immunophenotype/leukocyte subset distribution.

2. Constitutive activation status of T cell subsets.

3. T cell cytokine production profiles.

4. EBV-specific humoral responses.

5. EBV-direct plasma viral DNA via PCR.

6. Plasma cortisol levels.

## Results

### Field sample processing

**Figure 4:**
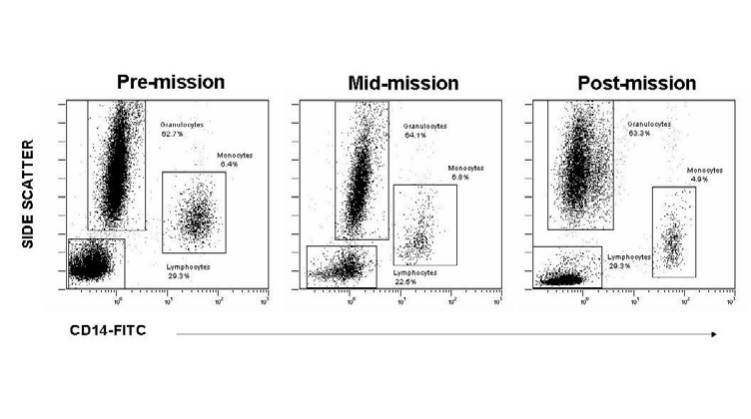
Representative flow cytometry scatter plots demonstrated the analytical integrity of the field-processed mid-mission samples. For flow cytometry analysis, whole blood was preserved with a non-cross linking preservative. This reagent was found to inhibit cellular auto fluorescence, as well as preserve antigenic structure of both extracellular as well as intracellular (cytokine) molecules. CD14 vs. side scatter plots allowing enumeration of granulocyte, monocyte and lymphocyte subsets are presented derived from a CD45+ gate to eliminate artifactual debris.

Cytometry revealed that the integrity of the mid-mission whole blood samples remained acceptable for analysis despite the in-field processing and the lengthy delay until analysis. This was true both for immunophenotype samples and cytometry analysis of the cells activated during culture. Although a mild elevation in green auto fluorescence was observed following analysis, all desired sub-populations could be resolved using multi-color and immunoscatter back-gating strategies to eliminate any artifactual debris (Figure 4). In addition, no observable degradation in sample was observed for any of the assays which used EDTA plasma as a sample (antibody levels, viral DNA, cortisol). Although it would be difficult to distinguish legitimate mid-mission changes from degraded sample for these assays, no degradation was expected, as field processing (removal of plasma) was not markedly different from standard laboratory processing. Also, the analytes for these assays are known to be stable for duration required prior to analysis. In contrast, the cytometry analysis of individual processed cells inherently allows an assessment of sample integrity during analysis.

### Peripheral immunophenotype

**Figure 5:**
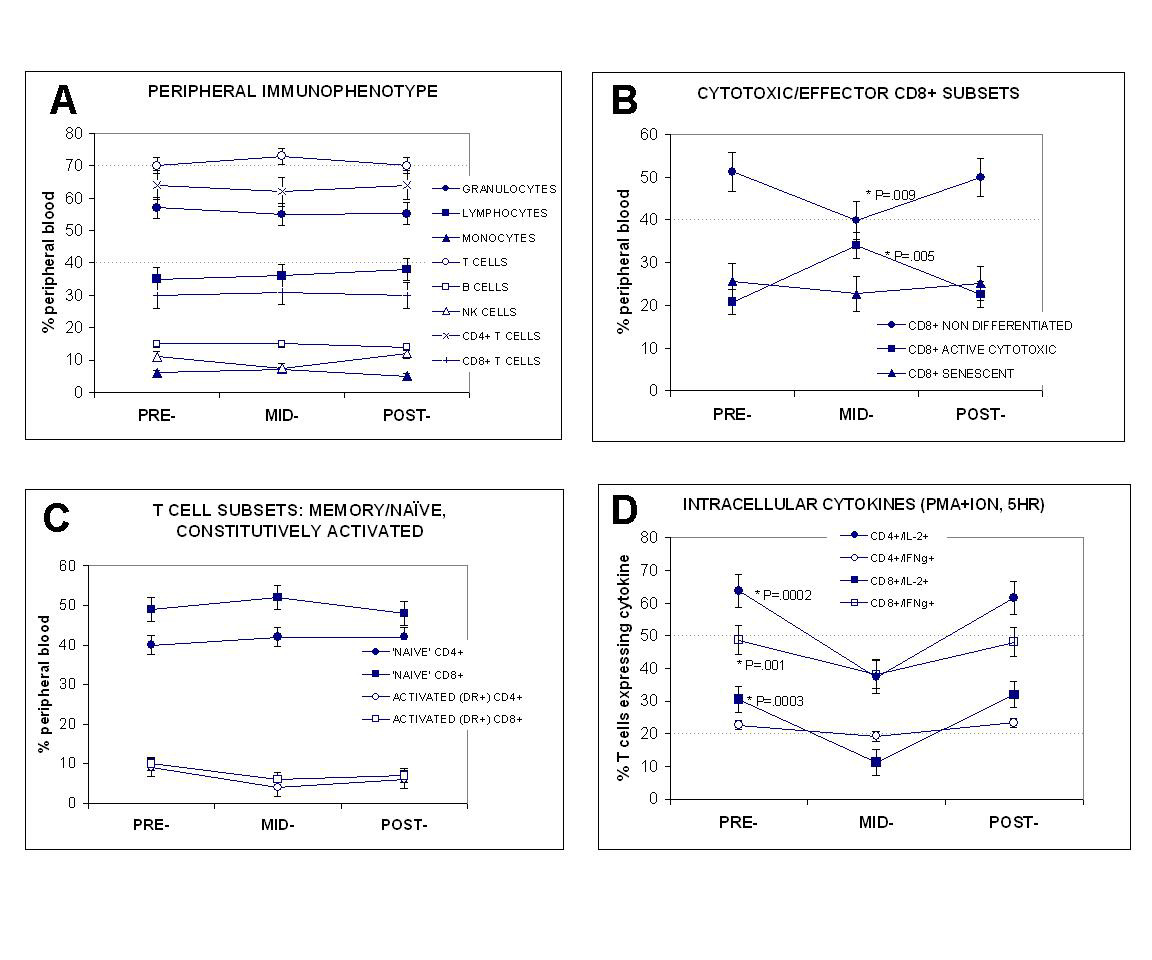
Mean peripheral blood percentages are plotted for (A) all major major leukocyte subsets; (B) cytotoxic, senescent and naïve (non-differentiated) CD8+ T cell subsets; and (C) memory (CD45RO+) and constitutively activated (HLA-DR+) CD4+ and CD8+ T cell subsets. (D) Mean percentages of T cell subsets capable of being stimulated to produce IFNg and IL-2. These data are derived from whole blood cultures stimulated with PMA+ionomycin for 5 hours in the presence of monensin to inhibit extracellular transport and allow intracellular accumulation. For all plots, mean values are shown without error bars. Data that were found to be statistically significant (pre-, vs. mid-) are indicated by '*' and the corresponding 'P' value. All other data were not statistically significant. Error bars represent the SEM for each point.

For the 10 HMP participants in this study, mean percentages of the leukocyte subsets, lymphocyte subsets and T cell subsets demonstrated no significant mid-mission alterations (figure 5a). When cytotoxic CD8+ T cell subsets were resolved based on the expression of CD28 and CD244 (C1.7), a significant increase in the active cytotoxic population (CD28+/CD244+) was observed, corresponding with a significant decrease in the non-differentiated population (CD28+/CD244-) (figure 5b). 'Major' memory and naïve T cell subsets were assessed based on the expression of cell surface CD45RA. There were no observable mid-mission differences in the levels of either naïve CD4+ T cells or naïve CD8+ T cells. There was also no mid-mission elevation in constitutively activated (HLA-DR+) CD4 or CD8 subsets. In fact, levels of constitutively activated T cells actually declined during the HMP mission, and were trending upwards towards baseline by R+60 (figure 5c).

### Intracellular cytokine analysis

The only functional assessment conducted during the HMP mission was an assessment of intracellular cytokine production by the CD4+ and CD8+ T cell subsets. These cultures were stimulated for 5 hours in the presence of PMA and ionomycin, with monensin added to block extracellular transport and allow intracellular accumulation. Compared to baseline, there was a significant decline in both the number of IL-2 producing CD4+ and CD8+T cell subsets mid mission that completely resolved by R+60 (figure 5d). The percentage of IFNg producing CD8+ T cells was also significantly reduced. The percentage of IFNg producing CD4+ T cells was reduced, but in a less dramatic fashion that did not achieve statistical significance. Levels of IFNg producing T cell subsets also did also resolve by R+60 (figure 5d). It is noteworthy that the cytokine alterations observed during the mission appeared to be cytokine-specific, and not specific to any particular T cell subset.

### Plasma viral antibody/DNA levels

To monitor the potential reactivation of latent EBV, levels of plasma EBV IgG and IgM were determined by immunofluorescence. Interestingly, one of the 10 HMP study participants was EBV sero-negative during pre-mission testing and appeared to remain sero-negative through the mid-mission assessment. Of the 9 EBV sero-positive individuals, 5 demonstrated an increased IgG titer mid-mission as compared to pre-mission values. However, for all five subjects the increase did not achieve what is usually associated with true reactivation of EBV (a four-fold increased titer). The remaining 4 individuals showed no change in IgG titer (Table 1). The presence of EBV IgM is associated with active or recent infection/reactivation, and the normal value is considered negative. One of the 9 participants was found to be IgM positive at the pre-mission assessment, and all 10 participants were IgM negative during mid-mission testing (Table 1). The assessment of plasma EBV DNA via PCR ('viral load') testing is also a sensitive indicator of active viral replication. All 10 of the study participants were found to be EBV DNA negative at both the pre-mission and mid-mission timepoints (table 1). Post mission testing was not performed for these parameters.

### Stress hormone levels

**Figure 6:**
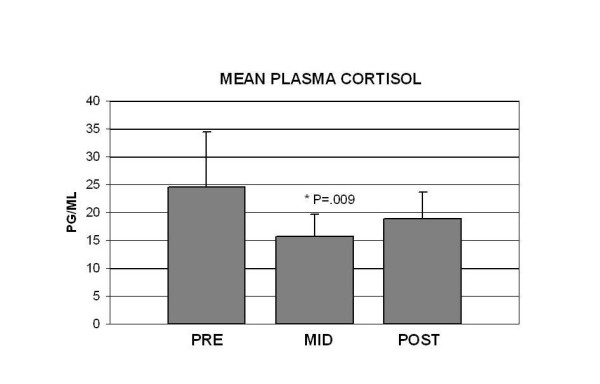
Mean pre-, mid-, and post-mission plasma cortisol levels for the HMP-2002 study participants.

Plasma cortisol was assessed in the HMP participants as a measure of physiological stress. Plasma cortisol is a relatively stable marker that was found to be compatible with the logistical limitations of the study, including sample storage and transport conditions. Compared to the pre-mission baseline assessment, there was a significant decrease in subject plasma cortisol mid-mission (mean levels 24.5 pg/ml, 15.6 pg/ml respectively; figure 6). Levels were trending towards recovery by R+60 (mean 18.9 pg/ml).

## Discussion

The purpose of this study was twofold: (1) to develop and validate a protocol for collecting, processing and stabilizing biological samples in remote field locations for immune assessment testing; and (2) to assess immune status in subjects participating in a simulated planetary exploration (spaceflight) analog. The data generated from the samples processed on-location demonstrate that a variety of field immunology laboratory techniques may be performed in remote settings without access to sophisticated equipment, provided transport to a laboratory for analysis may be arranged within approximately two weeks of sample processing. All whole blood cultures, immunophenotype, and plasma assays were successfully performed using the field-collected samples (figure 4). Immunophenotype was performed at JSC on whole blood samples that had been preserved on location. Although this protocol generally functioned well, lengthy delays in processing of sample tended to result in mildly elevated levels of green auto fluorescence. Using the cytometry techniques described above, this artifact did not adversely affect the data. Subsequent to this study however, the authors have found that on-location staining, lysing and fixation of all cytometry samples, followed by storage in 1.0% paraformaldehyde in PBS containing 1.0% non-fat dry milk significantly reduces the problematic auto fluorescence associated with storage in paraformaldehyde alone (data not shown). Samples stained for cytometry analysis in this manner and stored for up to two weeks are nearly indistinguishable from freshly stained samples. This is the technique currently employed by NASA when processing astronaut cytometry samples in Russia following landing of the International Space Station crews on the Russian Soyuz vehicle.

**Figure 7:**
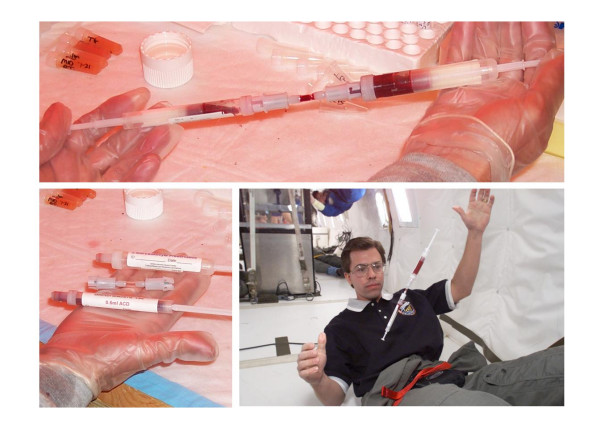
The Blood Collection/Preservation/Storage Apparatus (BCPSA) designed to safely preserve of whole blood samples during microgravity conditions. Whole blood is collected into a Monovette containing anticoagulant, and is transferred to a Monovette containing preservative via a novel interlink adaptor. Use of this device is highly advantageous during microgravity or field studies, as it greatly simplifies operations, reduces operator time and improves safety by nearly eliminating exposure to liquid blood samples. This device was validated during microgravity conditions onboard the NASA KC-135 parabolic flight aircraft in 2000, and utilized during the HMP 2002 immune study. Top and lower left: BCPSA in use at Devon Island; lower right: BCPSA evaluation during reduced gravity.

Given the epidemic disease rates in third-world countries, and the increasing sophistication of clinical laboratory equipment (limiting field use), we anticipate that protocols such as those validated here may have important future field research applications. In addition, protocols such as these could be modified for immune function testing on-orbit, to facilitate space physiology studies required prior to exploration-class space missions. Indeed, some of the microgravity-compatible hardware required to implement such investigations has already been developed. This study utilized the BCPSA for preservation of leukocytes (figure 7), and our laboratory previously described the Whole Blood Staining Device for on-orbit cell staining and whole blood culture [43,44]. Due to the many barriers to flying standard laboratory equipment in space (size, weight, liquid usage, power requirements, microgravity compatibility), a modified approach similar to the one described here could likely support immunology research investigations during low Earth orbit and short duration Lunar missions. Any routine monitoring during future long duration Lunar and Mars flights however, will likely require on-board analytical equipment, as timely sample return will be impossible.

Determining any clinical risk related to the effects of space flight on the human immune system presents several daunting challenges. First among these is a lack of in-flight data. The vast majority of flight immune studies have been pre-, and post-flight assessments. Unfortunately, post-flight assessments may be skewed by the effects of landing and readaptation to the 1 × G environment. Because of this, it is difficult to extrapolate post-flight data to the in-flight condition. Until comprehensive studies are initiated to assess in-flight immunity, it is useful to perform ground-based analog studies. The HMP represents a potentially good ground-based spaceflight analogs with regards to flight-associated immune dysfunction. Several likely causes of this phenomenon are shared between flight and HMP: long difficult travel, remoteness and isolation, extreme environment, disrupted circadian rhythms, and mission-associated stress. HMP participants keep rigorous schedules with respect to actual (not analog) exploration activities, hardware evaluation, and science studies, to maximize the benefit from their time on Devon Island. The only potential causes of flight immune dysregulation NOT shared with the HMP to some degree are high-energy radiation and microgravity. Interestingly, HMP participants are exposed to elevated levels of ultraviolet radiation due to the thinned atmospheric ozone concentrations in the Arctic [45], although it is not expected that short term elevated UV exposure would influence the parameters measured during this study.

The 10 HMP field participants and their environment had several important features in common with astronauts serving onboard the International Space Station. Both groups represent otherwise healthy and fit individuals performing mission activities in a remote and stressful environment. We believe that the data generated during this study accurately reflect what would be anticipated from such individuals. No consistent changes were observed in the distribution of any major peripheral leukocyte subsets. The leukocyte differential, lymphocyte subsets and T cell subsets were essentially unchanged between the pre-, mid-, and post-mission timepoints (figure 5a). In addition, no mid-mission changes were observed between the memory/naïve T cell subsets and no elevation occurred in the levels of constitutively activated (HLA-DR+) T cell subsets (figure 5b). Interestingly, the mean percentage of active cytotoxic (C1.7+/CD28+) CD8+ T cells did rise during the mission, corresponding to a decrease in the percentage of non-differentiated (C1.7-/CD28+) CD8+ T cells (figure 5c). The levels of these CD8+ T cell subsets resolved to baseline levels post-mission. The C1.7+/CD28+ subsets of CD8+ T cells is associated with higher cytotoxic activity and interferon-gamma production, and this subset is thought to control viral infections by mediating cytolytic activity against viral infected cells. As none of the participants demonstrated any mid-mission symptoms and no infections were reported, it is possible that the mid-mission associated rise in this subset is associated with latent viral reactivation or some other subclinical effect.

We believe that the immune subset data is consistent with otherwise healthy individuals in a stressful environment, and that these changes do not relate to the presence or absence of immune functional changes. Indeed, when a legitimate indicator of immune function was assessed, the cytokine profiles of the T cell subsets, sharp mission-associated decreases were observed. The ability of the T cell subsets (CD4+ and CD8+) to be stimulated to produce Interleukin-2 was significantly decreased mid-mission, as was the CD8+ population that could be stimulated to produce IFNg (figure 5d). All functional changes resolved back to near baseline values by the post-mission timepoint.

One consistent in-flight observation in astronauts is that the reactivation of latent herpes viruses occurs during spaceflight to high levels [19-22]. It is thought that the reactivation of these latent viruses may be a direct consequence to diminished immune function, as a healthy immune system is responsible for controlling these exacerbations. Latent viral reactivation is also known to occur during terrestrial stress situations. Plasma EBV antibody levels and direct viral DNA were assessed in the 10 HMP participants to determine if viral reactivation occurred during the mission. It is noteworthy that in the time following this study, better technology has evolved for monitoring viral specific immunity, such as tetramer quantitation of virus specific T cells, and peptide stimulation to monitor virus-specific T cell function [46]. In the HMP study subjects, 5 of 9 seropositve individuals did show an increase in plasma EBV IgG titers (Table 1). Although this could be an indicator of increased viral reactivation, none of the subjects were EBV IgM positive during the study, and none were positive for plasma EBV DNA. Based on this data, it cannot be said conclusively that any EBV reactivation occurred during the HMP mission. Future studies (incorporating assays such as tetramer staining and peptide stimulation) would be required to determine more precisely if low level latent viral reactivation occurs during HMP deployment.

It is well established that periods of physiological stress correlate with dysregulated immune function [47]. Classical dogma indicated that stress is immunosuppressive, via activation of the HPA axis and elevated levels of stress hormones. Corticosteroids have been demonstrated to suppress the function of immune cells, trafficking of leukocytes and cytokine production [47]. More recent studies however, show that certain types of acute stress may actually enhance immunity. Skin DTH responses are depressed following chronic stress, but are elevated following acute (2 h) stress [48]. In addition, the major stress hormones inhibit systemic IL-12, TNFa and IFNg (Th1 cytokines), but actually upregulate IL-10, IL-4 (Th2 cytokines) [49]. Thus, stress may be associated with a Th2 shift, postulated to protect the organism from 'overshooting' an inflammatory response. In fact, the neuro-immune interactions are extremely complex and specific effects depend largely on the type and duration of the stressful event.

Spaceflight itself may be a physiological stressor, and certainly landing, with a high-G entry and readaptation to the 1 × G environment after lengthy microgravity deconditioning, is a tremendous physiological stress. Following spaceflight, alterations in stress hormones have been observed in astronauts. Stowe et al. found that following spaceflight of 8–15 days there was no change in plasma cortisol or adrenocorticotropic hormone levels. In contrast, urinary epinephrine, norepinephrine and cortisol were significantly elevated following flight [18]. A more recent study found that mission duration had an effect on the levels of stress hormones following flight. After a 9 day mission, plasma cortisol was significantly decreased; however after a 16 day mission levels were significantly increased [31]. In contrast, urinary epinephrine and norepinephrine levels were greater after the shorter duration mission [31]. The conclusion was that shorter (acute) missions were characterized by a sympathetic response; whereas longer flight may be characterized by a glucocorticoid-mediated change related to longer microgravity duration. In the HMP participants, plasma cortisol was assessed as a measure of physiological stress. This hormone is stable enough following plasma purification, storage for the duration necessary, and sample return for subsequent analysis. The collection of 24-hour urine samples was unfortunately not considered realistic during the 2002 deployment, considering the other exploration activities being performed by the subjects. The significant decrease in plasma cortisol observed during the HMP mission (figure 6) is identical to the data derived from astronauts following 9 day spaceflight [31]. Considering the environmental conditions on Devon Island and the immune function data (figure 5d), it seems likely that the decreased plasma cortisol may be an indicator of the physiological stress related to deployment to Devon Island. These data may have correlated with an increased urinary cortisol level (had it been performed) and as observed in astronauts following the 16 day spaceflight [31]. The authors believe that reduced levels of plasma cortisol following a stressful event, in contrast to more persistent elevated urine levels, may represent a 'rebound' effect as the body re-adapts after the initial acute stress response. Essentially, the acute rise in plasma cortisol occurs rapidly after a stress event, and is quickly followed by recovery to baseline or even depressed levels. To derive a better understanding of the physiological stress aspects of this flight analog, it would be desirable to include a 24 hour urine collection and processing in any future HMP human physiology study.

Taken together, these data support the establishment of the HMP as a unique ground-based spaceflight/planetary exploration analog that may have significant utility for terrestrial human physiology studies. The HMP may be particularly useful for assessments of human physiology such as immunology, that are particularly influenced by stress, isolation and disrupted circadian rhythms.

## Methods

### Study design/subjects

This study protocol was designed to be analogous to spaceflight research: baseline testing was performed 30–60 days prior to the start of the mission to establish baseline values; mid-mission testing performed to assess mission-associated changes; and post-mission testing performed 30–60 days after return to confirm recovery to baseline values. Ten healthy male subjects scheduled to participate in the HMP-2002 field season were enrolled in this study. No female HMP 2002 participants were identified who could be solicited for this study, primarily for logistical reasons. The subjects were from a variety of locations and institutions located throughout the US and Canada (figure 1a). On Devon Island the subjects lived in standard cold weather tents (see figure 2) and generally worked long days participating in exploration activities. Subjects generally ate well, with meals provided by the HMP facility. Approval from the Johnson Space Center Committee for the Protection of Human Subjects (CPHS) was obtained prior to the initiation of the study. Informed consent was obtained from each subject prior to participation in the study.

### Whole blood samples

Whole blood samples consisted of 10.0 ml EDTA and 10.0 ml heparin whole blood collected per subject per timepoint using standard phlebotomy techniques during the morning hours. Because it was necessary to collect pre- and post-mission samples near the local residence of the subject, clinical laboratories from local hospitals were enlisted to perform the pre- and post-mission sample collections. Pre- and post-mission samples were shipped overnight to JSC for analysis. Previous in-house validation has shown that a 24-hour delay in sample processing did not affect the results for the parameters measured during this study (data not shown). For consistency of data, all mid-mission blood samples collected on Devon Island were held for 24 hours in the laboratory tent at ambient temperature (heated, approximately 15–22 degrees C) prior to processing to duplicate the pre- and post-mission sample shipping delays. Specific sample collection points for each subject are presented in figure 3.

### Immunophenotype analysis

A 5-color flow cytometry antibody matrix was created (using FITC, PE, PE-Texas Red, PC5 and APC as fluorochromes) that assessed most of the major leukocyte/lymphocyte subsets, as well as various memory/naïve and activated T cell subsets (see table 2). All labeled antibodies were obtained from Beckman Coulter Corporation, Miami, Florida, or Becton Dickinson, Franklin Lakes, NJ, and were used according to the manufacturer's instructions. Cell surface markers were stained first combining 100 ul of EDTA whole blood and 10 μg of each appropriate labeled monoclonal antibody. Staining was performed by incubated the mixture at room temperature for 20 minutes. Red blood cells were lysed using Beckman-Coulter optilyse as described by the manufacturer. Stained leukocytes were then fixed in 1.0% paraformaldehyde in PBS for 10 minutes prior to analysis. Analysis was performed on a Beckman-Coulter ALTRA Cytometer configured for 5 color analysis using argon and HeNe dual lasers. All common leukocyte sub populations were analyzed: granulocytes, lymphocytes, monocytes, T cell CD4/CD8 subsets, effector/cytotoxic CD8+ subsets, memory/naïve (CD45RA-/+) and activated T cell subsets (HLA-DR+). Modifications to this procedure during activities on Devon Island are described below.

### Intracellular cytokine analysis

Lymphocyte intracellular cytokine profiles were assessed for specific cell subsets at the single-cell level utilizing intracellular flow cytometry. The unique advantage of this technique is the ability to assess the production of multiple cytokines simultaneously in positively identified cell sub-populations using multi-color flow analysis. Whole blood culture was performed to eliminate the need for cell purification steps [43]. Also, whole blood culture may also represent more accurately in-vivo responses since all cell types are present to preserve cell-cell reactions, as well as any soluble factors present in the plasma. Cultures were set up by adding 100 ul of heparinized whole blood to 1.0 ml of RPMI culture media. A combination of PMA (10 ng/ml) and Ionomycin (10 ug/ml) was used for T cell stimulation with monensin (3.0 uM) added to the cultures to halt the extracellular transport of cytokines and allow intracellular accumulation to detectable levels. Cultures were incubated for 5 hours at 37 degrees Centigrade. Following culture, the activated blood was washed, supernatants were removed, the RBCs lysed as noted above, and the remaining WBCs were fixed in 4.0% paraformaldehyde for 10 minutes. To detect intracellular production of IFNγ or IL-2 (following surface marker staining), the fixed PBMC's were resuspended in 200 μl of permeabilization buffer, consisting of 5.0% non-fat dry milk and 0.5% saponin in PBS to which 0.5 μg of labeled mouse antibody to either IFNγ or IL-2 (or both) was added. The cells were incubated at room temperature for 25 minutes and then washed in PBS containing saponin. The cells were then resuspended in 1.0% paraformaldehyde for analysis.

### EBV antibody levels

EBV VCA IgG testing was performed using the Merifluor EBV IgG IFA kit (Meridian Bioscience, Inc., Cincinnati, OH) according to the manufacturer's instructions. EBV IgM antibody was detected using the Diamedix EBV VCA IgM assay (Diamedix, Inc, Miami, FL) according to the manufacturer's instructions on the MAGO automated microplate robotic system (Diamedix).

### EBV plasma viral load

EBV PCR was performed by real time PCR using primers/probe set first described by Kimura, H. et al. [50]. 200 microliters of whole blood was processed using the Qiagen Blood DNA kit according to the manufacturer's instructions. Five microliters of the eluted DNA was added to a PCR master mix prepared using the TaqMan Universal PCR Master Mix, no AmpErase UNG (Applied Biosystems, Foster City, CA), uracil-N-glycosylase (UNG) from Epicentre Inc. and the EBV specific primers (100 nmolar) and probe (200 nmolar). Real time PCR was performed using an ABI 7000 SDS (Applied Biosystems) with cycling conditions of 37 degrees C for 10 minutes, 95 degrees C for 10 minutes and 50 cycles of 95 degrees for 15 seconds and 60 degrees C for 60 seconds. Sensitivity of the PCR was determined to be 5 input copies which equates to approximately 250 copies per ml of whole blood.

### Plasma cortisol levels

The measurement of plasma cortisol was performed by enzyme-linked immunosorbant assay (Diagnostic Systems Laboratories, Webster, Texas) as has previously been described in detail [18,51].

### Field sample processing protocol

No laboratory equipment was transported for real-time field analysis. Instead, techniques were developed allowing field culture, processing and preservation of samples for subsequent laboratory analysis after mission completion. In general, the field activities consisted of the following activities:

• Collection of whole blood samples.

• Setup of whole blood culture for T cell activation.

• Preservation of whole blood for flow cytometry assays.

• Preservation of activated leukocytes for intracellular cytokine detection.

• Purification and aliquoting of plasma for stress hormone and viral assays.

The sample processing performed on Devon Island rendered all sample types stable for storage and transport to JSC for subsequent analysis. During the deployment, processing was aided by the development of novel hardware for processing blood samples, the use of a newly developed commercial leukocyte preservatives and the development of field sample processing techniques. In most cases, processing rendered the samples stable for up to two weeks without cold storage allowing easy transport off Devon Island for subsequent analysis at Johnson Space Center. The individual modifications for field use per assay are as follows:

### Field blood collection

On Devon Island, a phlebotomy station was set up in the main laboratory tent. Samples were collected from each subject by venipuncture using sterile techniques and butterfly type collection equipment. This does not represent a significant deviation from standard blood collection techniques.

### Field leukocyte subset analysis

Whole blood was treated with a recently developed commercial preservative (Cytochex, Streck Laboratories Inc.) [52] that maintains the antigenic structure of the cell surface molecules for at least 7–31 days [53,54]. For this study our laboratory validated that samples may be stored chilled for up to two weeks with out loss of subset resolution by flow cytometry (data not shown). Representative scatter plots demonstrating no loss of subset discrimination are presented in figure 4. Typical long-term storage of paraformaldehyde fixed samples for flow cytometry analysis (> 2 days) results in a marked increase in green auto-fluorescence that may hinder analysis. The Cytochex preservative is non-crosslinking, which was found to greatly reduce the problematic autofluorescence associated with samples stored long term in only paraformaldehyde. There are however several markers that are largely or totally incompatible with this preservative. CD19 staining is diminished, and CD62L is rendered unstainable following Cytochex treatment. For markers such as these, the only option is to actually stain the cell samples in standard fashion and preserve until analysis.

### Field blood preservation hardware

To facilitate field sample processing, and to reduce the amount of biohazardous waste, whole blood preservation was performed on Devon Island using the Blood Collection/Preservation/Storage Apparatus (BCPSA) (Figure 7). This device consists of a Monovette preloaded with preservative, a second anticoagulated Monovette for blood sample collection, and a novel interlink adapter. Via connection of the interlink adaptor, it is possible to quickly and easily transfer whole blood into the liquid preservative. The adaptor is then removed and discarded, and the storage vessel stem is removed, creating a stable long-term storage environment for the preserved blood. The BCPSA functions in a completely microgravity-compatible fashion, and was developed for potential future sample processing onboard the International Space Station. Microgravity validation of this device was performed onboard the NASA KC-135 reduced gravity aircraft in 2000 (Figure 7). The BCPSA would be advantageous for use in other field clinical studies, particularly those involving infectious disease, where it is desirous not to manually pipette potentially infectious blood samples. Use of the BCPSA completely protects the user from accidental exposure to the liquid blood sample during processing.

### Field cell culture protocol

All cell culture reagents were transported to Devon Island chilled and stored chilled until use. Previous in-house testing has validated the stability of all the cell culture mitogens (PMA, ionomycin, monensin) without loss of activity for up to two weeks when stored in this manner (data not shown). Typical laboratory incubators supply two items to the incubating cells: 37 degree heat, and CO_2_. Since short term culture in buffered media does not require exogenous CO2, whole blood cultures were performed on Devon Island in 12 × 16 mm polystyrene tubes that were tightly sealed. A similar method for whole blood culture in portable devices was previously described [43]. A portable field incubator was developed consisting of a small soft-side insulated box and a heating coil capable of holding 37°C. Temperature was monitored throughout the incubation and did not deviate +/- 2 degrees from 37°C. Finally, we have found that the performance of short term cultures such as these precludes the need to set up the cultures in a biological safety cabinet for sterility (for most assays). Prior to the HMP mission, parallel studies found that intracellular cytokine cultures and analysis setup using standard laboratory techniques (CO2 exposure, sterile hood, etc.) and the field technique were indistinguishable (data not shown). Following culture, the supernatant was removed and the activated cells were preserved by the addition of 500 ul of Cytochex preservative for transport to JSC. Previous in-lab validation had demonstrated that this reagent preserved the intracellular cytokines as well as the surface antigens at room temperature without the need for cold storage. Preserved activated cell samples were stored either at room temperature or 4 degrees 37°C during storage on-location or during transport to JSC for analysis.

### Field plasma separation and storage

To purify and aliquot EDTA plasma on-location at Devon Island for viral and cortisol assays, a small centrifuge was transported to the island. EDTA whole blood was centrifuged for 10 minutes, and plasma was removed and stored in labeled cryo-vials. Plasma samples were stored at 4–5°C when possible, and briefly at room temperature otherwise (during air travel), for transport to JSC for analysis. Salivary cortisol has been found to be extremely stable when stored at 5°C, with values unchanged following storage of up to 3 months [55]. We have validated that plasma cortisol is stable at room temperature for up to 2 weeks (data not shown).

### Statistical analysis

To determine mission-associated alterations, mid-mission data were compared to pre-mission baseline values. Statistical operations were performed by using Microsoft Excel 2003. The Student's paired *t *test was used and p values of less than 0.05 were considered significant. Where appropriate, significant data is indicated by '*'.

## Competing interests

The author(s) declare that they have no competing interests.

## Authors' contributions

BC, CS and PL participated in conception of this study. BC performed all field sample processing, all flow cytometry assessments, all data analysis and drafted the manuscript. PL was responsible for all HMP 2002 logistical concerns, and serves as principal investigator of HMP. RS performed all hormone assessments, contributed to the manuscript draft. JJ and RE are NASA flight surgeons and HMP veterans; they contributed to the conception of the study, aided clinical interpretation of the data and contributed to the manuscript draft. RW was responsible for all viral assays and provided inputs to the manuscript. CS is principal investigator of the NASA-JSC Immunology Laboratory, where majority of testing performed, and provided inputs to the manuscript. All authors have read and approved the final version of the manuscript.

## Figures and Tables

**Table 1:** Subject plasma levels of EBV IgG, EBV IgM and EBV DNA before and during HMP deployment.

	**EBV IgG**		**EBV IgM**		**EBV DNA**	
**SUBJECT**	**PRE**	**MID**	**PRE**	**MID**	**PRE**	**MID**
1	640	640	NEG	NEG	NEG	NEG
2	320	**640**	NEG	NEG	NEG	NEG
3	2560	2560	NEG	NEG	NEG	NEG
4	0	0	NEG	NEG	NEG	NEG
5	320	**640**	**POS**	NEG	NEG	NEG
6	640	**1280**	NEG	NEG	NEG	NEG
7	1280	1280	NEG	NEG	NEG	NEG
8	640	**1280**	NEG	NEG	NEG	NEG
9	1280	1280	NEG	NEG	NEG	NEG
10	640	**1280**	NEG	NEG	NEG	NEG

Antibody levels were determined by immunofluorescence, DNA levels were assessed by PCR. Plasma antibody levels are expressed as titer. Post mission assessments were not performed.

**Table 2:** Fluorescent antibody panel for the 2002 Haughton Mars Immune Assessment

**FITC**	**PE**	**ECD**	**PE/Cy5**	**APC**	**CELL POPULATIONS**
CD14 (RMO52)			CD45 (J.33)		WBC differential: granulocytes/lymphocytes/monocytes
CD3 (UCHT1)	CD16 (3G8)	CD19 (J4.119)	CD45 (J.33)		Lymphocyte subsets: T cells, B cells and NK cells
CD4 (13B8.2)	CD8 (B9.11)		CD3 (UCHT1)		T cell subsets: CD4+ vs. CD8+
CD45RA (ALB11)	CD45RO (UCHL1)		CD4 (13B8.2)	CD3 (UCHT1)	Naïve/memory CD4+ T cell subsets
CD45RA (ALB11)	CD45RO (UCHL1)		CD8 (B9.11)	CD3 (UCHT1)	Naïve/memory CD8+ T cell subsets
CD8 (B9.11)	CD28 (CD28.2)		CD244 (C1.7)	CD3 (UCHT1)	Cytotoxic effector/suppressor CD8+ T cell subsets
HLA-DR (Immu-357)	CD69 (TP1.55.3)	CD8 (B9.11)	CD4 (13B8.2)	CD3 (UCHT1)	Activation status of T cell subsets (CD4/CD8+)
IFNg* (5344.111)	IL-2* (25723.11)	CD8 (B9.11)	CD3 (UCHT1)		Intracellular detection of cytokines in T cell subsets

All antibodies purchased from either the Beckman-Coulter Corporation except for (*), purchased from  Dickinson. The specific antibody clones are indicated in parentheses.
